# Increasing TIMP3 expression by hypomethylating agents diminishes soluble MICA, MICB and ULBP2 shedding in acute myeloid leukemia, facilitating NK cell-mediated immune recognition

**DOI:** 10.18632/oncotarget.16657

**Published:** 2017-03-29

**Authors:** Aroa Baragaño Raneros, Alfredo Minguela Puras, Ramon M. Rodriguez, Enrique Colado, Teresa Bernal, Eduardo Anguita, Adela Vasco Mogorron, Alberto Chaparro Gil, Jose Ramon Vidal-Castiñeira, Leonardo Márquez-Kisinousky, Paula Díaz Bulnes, Amelia Martinez Marin, García Maria Carmen Garay, Beatriz Suarez-Alvarez, Carlos Lopez-Larrea

**Affiliations:** ^1^ Department of Immunology, Hospital Universitario Central de Asturias, Oviedo, Spain; ^2^ Immunology Service, Instituto Murciano de Investigación Biosanitaria (IMIB), Hospital Clínico Universitario Virgen de la Arrixaca, Murcia, Spain; ^3^ Department of Hematology, Hospital Universitario Central de Asturias, Oviedo, Spain; ^4^ Hematology Department, Hospital Clínico San Carlos, Instituto de Investigación Sanitaria San Carlos (IdISSC), Department of Medicine, Universidad Complutense de Madrid (UCM), Madrid, Spain; ^5^ Hematology Service, Hospital Clínico Universitario Virgen de la Arrixaca, Murcia, Spain; ^6^ Hematology Service, Hospital General Universitario Santa Lucía, Cartagena, Murcia, Spain

**Keywords:** acute myeloid leukemia (AML), DNA methylation, NKG2DL, NKG2D, TIMP3

## Abstract

Acute myeloid leukemia (AML) is a disease with great morphological and genetic heterogeneity, which complicates its prognosis and treatment. The hypomethylating agents azacitidine (Vidaza^®^, AZA) and decitabine (Dacogen^®^, DAC) have been approved for the treatment of AML patients, but their mechanisms of action are poorly understood. Natural killer (NK) cells play an important role in the recognition of AML blasts through the interaction of the activating NKG2D receptor with its ligands (NKG2DL: MICA/B and ULBPs1-3). However, soluble NKG2DL (sNKG2DL) can be released from the cell surface, impairing immune recognition. Here, we examined whether hypomethylating agents modulate the release of sNKG2DL from AML cells. Results demonstrated that AZA- and DAC-treated AML cells reduce the release of sNKG2DL, preventing downregulation of NKG2D receptor on the cell surface and promoting immune recognition mediated by NKG2D-NKG2DL engagement. We show that the shedding of MICA, MICB and ULBP2 is inhibited by the increased expression of TIMP3, an ADAM17 inhibitor, after DAC treatment. The *TIMP3* gene is highly methylated in AML cells lines and in AML patients (25.5%), in which it is significantly associated with an adverse cytogenetic prognosis of the disease. Overall, TIMP3 could be a target of the demethylating treatments in AML patients, leading to a decrease in MICA, MICB and ULBP2 shedding and the enhancement of the lytic activity of NK cells through the immune recognition mediated by the NKG2D receptor.

## INTRODUCTION

Acute myeloid leukemia (AML) is characterized by the accumulation of myeloid precursors in bone marrow and peripheral blood [1]. AML patients were originally categorized by the French-American-British (FAB) classification on the basis of their cellular morphology. Since 2008, and extended in 2016, the World Health Organization (WHO) has additionally characterized AML patients with respect to specific genetic abnormalities [2]. Both classifications reveal the considerable clinical and biological heterogeneity of this disease, but the therapeutic options remains very limited. Genome-wide epigenetic studies have shown that mutations in genes involved in DNA methylation (DNMT3A, TET2 and IDH) or aging-associated changes are the main causes of the aberrant DNA methylation pattern noted in AML patients [3]. DNA hypomethylating agents are currently approved by the US Food and Drug Administration (FDA) for the treatment of patients with AML and myelodysplastic syndromes. Azacytidine (Vidaza^®^, AZA) and decitabine (Dacogen^®^, DAC) are cytidine nucleoside analogs whose main advantage over conventional care regimens is less toxicity of the drug, better tolerability and better overall survival of AML patients [4–6]. These hypomethylating drugs affect cell viability, cell cycle, and changes in gene expression profiles [7]. Thus, knowing the molecular patterns that are modulated by these agents could be essential to improving their effectiveness and for identifying new biomarkers that predict the response to these drugs.

Natural killer (NK) cells play an important role in the innate immune response to AML through a balance between activating and inhibiting signals [8]. NKG2D is one of the most important activating receptors expressed in CD8^+^ T cells, NK cells, γδ T cells and NKT cells [9]. NKG2D recognizes two families of stress-inducible ligands (NKG2DL): the MHC class I-related molecules (MICA/MICB) and the UL-16-binding proteins (ULBPs 1-6) [10, 11]. The expression of NKG2DL in AML cells is highly heterogeneous and controversial. Some authors have shown that most AML patients express at least one NKG2DL at the cell surface [12], whilst others have reported low or null expression levels of these ligands in AML cells [13, 14]. In addition, AML cells have developed several regulatory mechanisms to downregulate NKG2DL expression and avoid NKG2D-mediated immunosurveillance. Epigenetic modifications have been proposed as being key mechanisms for modulating NKG2DL expression in AML cells. Treatment with valproic acid, a histone deacetylase inhibitor, enhances MICA, MICB and ULBP1 surface expression in AML blasts, increasing the killing activity of NK cells [15]. In this way, we have previously reported that *MICA*, *ULBP1*, *ULBP2* and *ULBP3* genes are aberrantly hypermethylated in AML cells, and that treatment with demethylating agents increases their expression promoting recognition and cytolysis by NK cells [16]. Moreover, NKG2DL can also be released from the surface of tumor cells, leading to downregulation of their NKG2D receptor and damaging their recognition by cytotoxic NKG2D-positive cells [17]. Some NKG2DL are more susceptible to metalloprotease (MP) cleavage and to release as soluble proteins, whilst other NKG2DL are recruited to exosomes [18–23]. The matrix metalloproteases (MMPs) MMP9 and MM14, and the ADAM (a disintegrin and metalloproteinase) family (ADAM9, ADAM10 and ADAM17, also known as TACE) are mainly known for their involvement in NKG2DL cleavage, and some, such as ADAM17, can be found in exosomes [24]. Thus, the different mechanisms of release for NKG2DL could depend on the cell type, the cellular metabolism, and even the availability of MMPs [25].

The tissue inhibitor of metalloproteinases-3 (TIMP3), a potent inhibitor of the MMP subfamily and some ADAMs, has been associated with MICA and MICB shedding [26, 27]. The presence of high levels of sNKG2DL in the serum of AML patients has been associated with poor survival and lower complete remission rates [12, 28]. Therefore, a detailed knowledge of the mechanisms involved in the regulation of sNKG2DL could usefully be applied to prevent the immune escape of tumor cells.

In this study, we analyze the effect of hypomethylating agents on the shedding of sNKG2DL in AML cells and their consequences for NK cell-mediated immune recognition. We show that (i) AZA and DAC limit the release of all NKG2DL in the supernatants of AML cell lines; (ii) decreased levels of sNKG2DL prevent the downregulation of the NKG2D receptor and favor the recognition and lysis of AML cells by NKL cells; (iii) ADAM17 is the sheddase involved in the release of sNKG2DL in AML cell lines; (iv) demethylation of *TIMP3* gene may be responsible for the lower level of shedding of MICA, MICB and ULBP2 in AML cells; and (v) high TIMP3 DNA methylation levels in AML patients are associated with an adverse cytogenetic prognosis for the disease. Therefore, our study reveals that hypomethylating treatments in AML cells could modulate the shedding of MICA, MICB and ULBP2 in a TIMP3 demethylation-dependent manner.

## RESULTS

### Hypomethylating treatments limit NKG2DL release, promoting NKG2D-mediated NKL cell recognition

**Figure 1:**
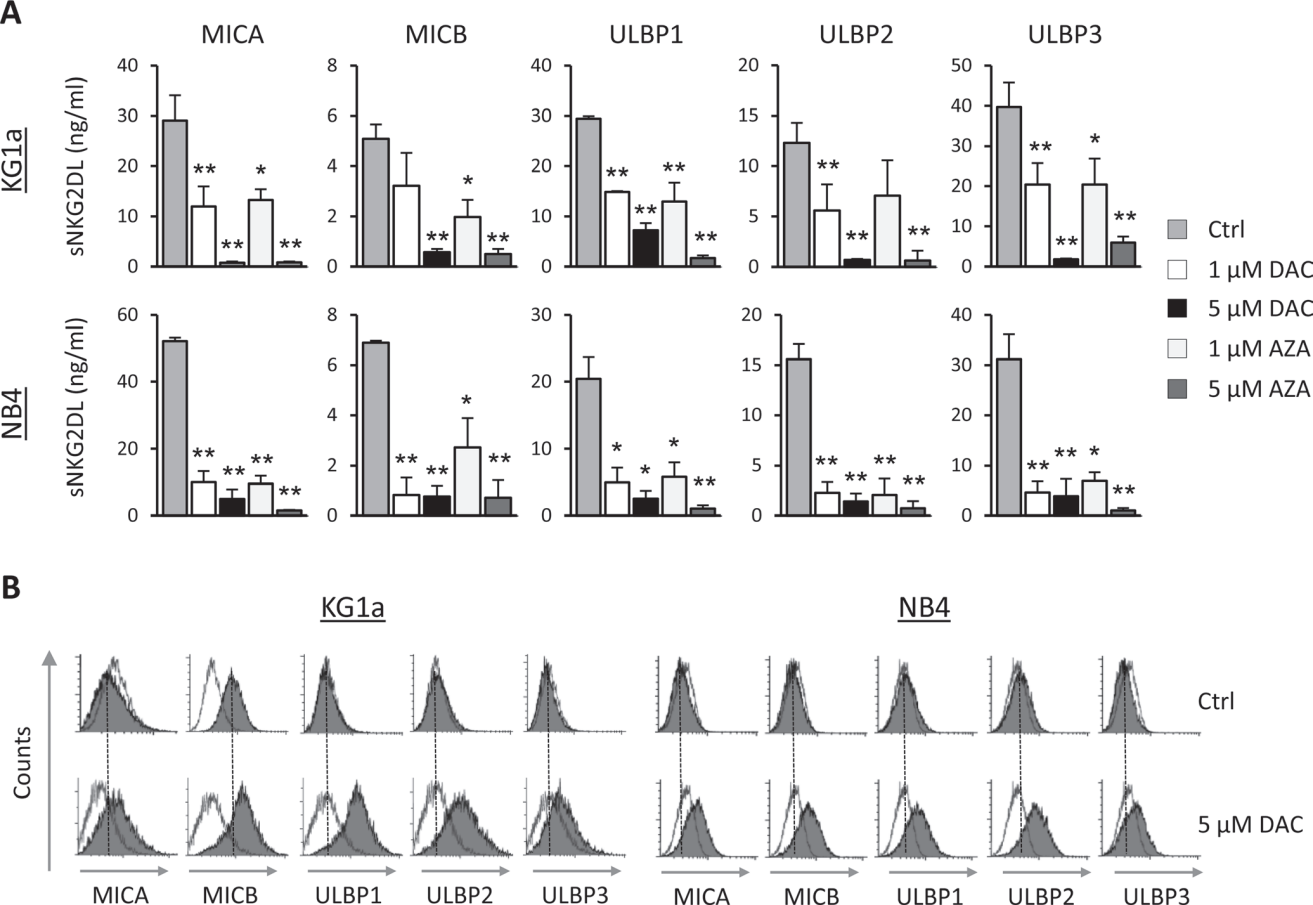
Hypomethylating agents (DAC and AZA) decreases the release of sNKG2DL, inducing their expression on the surface of AML cells (**A**) AML cell lines (KG1a and NB4 cells) were treated with DMSO (Ctrl), DAC (1 and 5 μM) or AZA (1 and 5 μM) for 48 hours. Soluble levels of MICA, MICB, ULBP1, ULBP2 and ULBP3 ligands were quantified by sandwich ELISA. Each bar represents the mean ± SEM of at least four independent experiments. *p < 0.05 and ^*^p < 0.001. (**B**) KG1a and NB4 cells were treated with 5 μM of DAC for 48 hours. Before and after treatment, cell surface expression of NKG2DL was analyzed by flow cytometry using monoclonal antibodies specific to each NKG2DL (MICA, MICB, ULBP1, ULBP2 and ULBP3), followed by incubation with FITC-conjugated goat anti-mouse IgG. The white histograms represent the isotype control antibody and shaded grey histograms show the NKG2DL expression. Dotted vertical lines indicate mean fluorescence intensity value in untreated samples.

We determined the effect of the AZA and DAC hypomethylating agents on the release of sNKG2DL (MICA, MICB, ULBPs1-3) in two AML cell lines (KG1a and NB4) that showed high levels of these soluble molecules in their cellular supernatants at basal level. AML cells were treated with DAC or AZA (1 μM or 5 μM) for 48 hours, and the presence of sNKG2DL in the cell-free supernatants was quantified by ELISA. The levels of all sNKG2DL were significantly reduced after treatment with both demethylating drugs (Figure 1A). The downregulation was dose-dependent, but the pattern was not identical in the two cell lines, the difference was more pronounced at 1 μM in the NB4 cell line than in the KG1a cells. However, in both cell lines, all sNKG2DL were reduced by more than 90% at the highest doses. As similar effects were obtained with both hypomethylating agents and because DAC treatment is less toxic to the AML cells, further *in vitro* experiments were carried out with DAC alone. Moreover, as shown in Figure 1B, reduced release of sNKG2DL after DAC treatment was associated with the increased expression of these ligands on the surface of AML cells.

**Figure 2:**
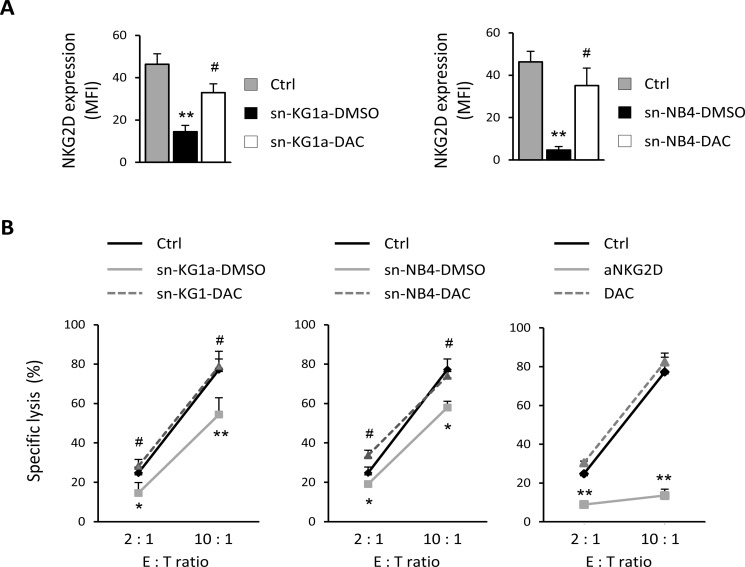
Reduced shedding of sNKG2DL after DAC treatment restores the NKG2D expression and favors the immune recognition mediated by NKG2D-NKG2DL engagement (**A**) NKL cells were co-cultured with cell-free supernatants (sn) obtained from KG1a and NB4 cells untreated (sn-DMSO) or treated with 1 μM DAC for 48 hours (sn-DAC). NKL cells grow in culture medium were considered as a control (Ctrl). NKG2D expression was analyzed by flow cytometry and represented as mean fluorescence intensity (MFI). Each bar represents the mean ± SEM of three independent experiments. ^*^ versus control and *p* < 0.01; ^#^ versus sn-DMSO and *p* < 0.05. (**B**) NKL cells were co-cultured with K562 cells at the indicated E:T ratio in a cell lysis assay, in the absence (Ctrl) or presence of cellular supernatant derived from KG1a (left panel) and NB4 (middle panel) cells previously treated with DMSO (sn-DMSO) or 1 μM DAC (sn-DAC) for 48 hours. Specificity of the NKG2D-NKG2DL interaction was corroborated using an anti-NKG2D blocking mAb and the effect of DAC was assayed to analyze the non-specific effects on the lytic capacity of NKL cells (right panel). Measurements were made in duplicate and the mean ± SEM of the two independent experiments are shown. * versus control and *p* < 0.05; ^*^ versus control and *p* < 0.01; ^#^ versus sn-DMSO and *p* < 0.05.

The presence of NKG2DL in soluble forms can have a dual effect: on the one hand, it down-modulates the NKG2D receptor on the surface of NKG2D-positive effector cells, and on the other hand, it damages the immune recognition mediated by cytotoxic cells by blocking the NKG2D-binding site to their NKG2DL expressed on the surface of target cells. To explore those functional consequences after DAC treatment, we first analyzed the effect of sNKG2DL on the expression of NKG2D receptor in NKL cells before and after DAC treatment. NKG2D expression was significantly weaker in the presence of supernatants from untreated AML cells (sNKG2DL-positive; *p* = 0.009 for KG1a and *p* = 0.002 for NB4) whilst the NKG2D expression was partially restored after incubation with DAC-treated supernatants (*p* = 0.023 for KG1a and *p* = 0.011 for NB4) (Figure 2A). Additionally, we assayed the effect of sNKG2DL on the blockage of the NKG2D-NKG2DL engagement. To do this, we carried out cytotoxicity assays using the NKL cell line, which expresses high levels of NKG2D receptor, as a source of effector cells, and, as target cells, the human leukemic cell line K562, which expresses high levels of NKG2DL on the cell surface (data not shown). Effector (E) and target (T) cells were co-cultured, at different E:T ratios, in the presence of cell-free supernatants obtained from untreated and DAC-treated AML cells. As shown in Figure 2B (right panel), immune recognition was dependent on the NKG2D-NKG2DL interaction because blocking NKG2D with an anti-NKG2D monoclonal antibody (mAb) completely inhibited specific lysis. Moreover, no effects on lysis were observed when exogenous DAC was added under basal conditions, confirming that the DAC lack of non-specific effects on the lytic capacity of NKL cells (Figure 2B, right panel). So, when the cytotoxicity assay was performed in the presence of supernatants (sNKG2DL-positive) from untreated-AML cells (KG1a and NB4), the lytic ability of NKL cells was significantly reduced at all assayed E:T ratios (*p* = 0.0045 for KG1a and *p* = 0.014 for NB4 at 10:1 E:T ratio) (Figure 2B, left and middle panel). However, in the presence of supernatants obtained from DAC-treated AML cells (reduced sNKG2DL), the specific lysis was similar to basal conditions and significantly greater than in supernatants from untreated AML cells (*p* = 0.0016 for KG1a and *p* = 0.026 for NB4 at 10:1 E:T ratio).

Thus, our results suggest that downregulation of sNKG2DL in AML cells after DAC treatment favors NKL cell-mediated immune recognition through NKG2D-NKG2DL engagement.

### ADAM17, but not ADAM10, is involved in NKG2DL shedding by AML cells

**Figure 3:**
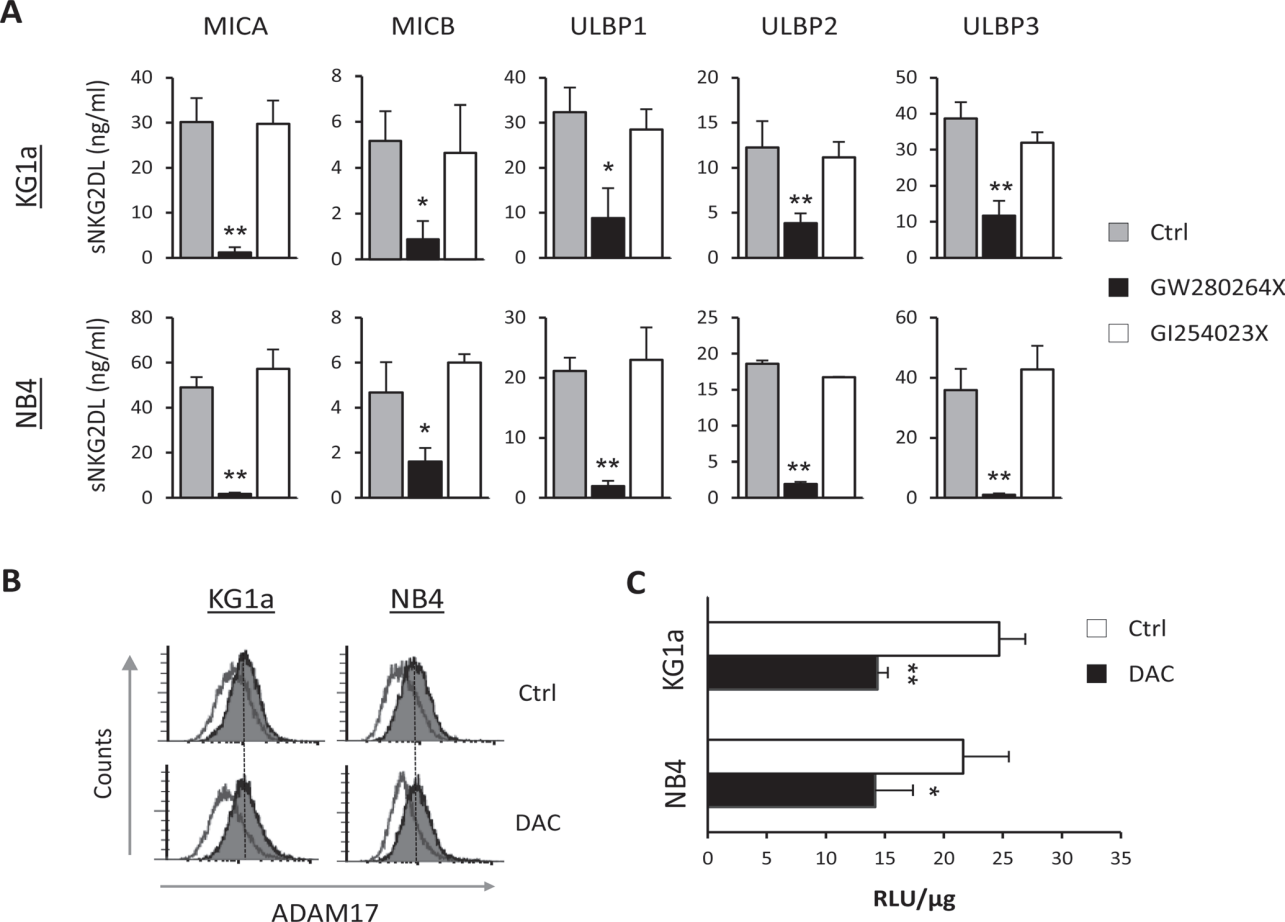
ADAM17, but not ADAM10, regulates NKG2DL shedding in AML cell lines (**A**) KG1a and NB4 cells were treated with inhibitors specific to ADAM17 (10 μM GW280264X) and ADAM10 (50 μM of GI254023X) for 48 hours. Levels of soluble NKG2DL (sMICA/B and sULBPs1-3) were quantified by sandwich ELISA. Values are the mean ± SEM of at least three independent experiments. *p < 0.05 and ^*^p < 0.01. (**B**) KG1a and NB4 cells were treated with DMSO (Ctrl) or DAC (1 μM) for 48 hours. After treatment, cell surface expression of ADAM17 was analyzed by flow cytometry using anti-human ADAM17 monoclonal antibody, followed by incubation with FITC-conjugated goat anti-mouse IgG. The white histograms represent the isotype control antibody and the shaded grey histograms show the ADAM17 expression. (**C**) ADAM17 activity in KG1a and NB4 cells was measured in whole-cell lysates after treatment with DMSO (Ctrl) or DAC (5 μM) for 48 hours. Data are expressed as relative fluorescence units (RLU) at Ex/Em=490/520 nm absorbance normalized with respect to micrograms of total protein (RLU/μg). Values are the mean ± SEM of three independent experiments. *p < 0.05 and ^*^p < 0.01.

Proteolytic shedding of NKG2DL is mediated by different proteases in which ADAM10 and ADAM17 play a key role [17]. We analyzed the involvement of these two proteases in the release of sNKG2DL by AML cells. KG1a and NB4 cells were treated for 48 hours with specific inhibitors for ADAM17 (GW280264X) and ADAM10 (GI254023X), and the presence of sNKG2DL was quantified by ELISA in the culture supernatants. Pharmacological inhibition of ADAM17 produced a significant decrease in all sNKG2DL levels in both AML cell lines but no change was observed after ADAM10 inhibition (Figure 3A). Similar results were observed with TMI-1, an inhibitor of ADAM17 and other MMPs, but not of ADAM10 activity (Supplementary Figure 1). These results suggest that only ADAM17 is mainly involved in the shedding of NKG2DL in AML cell lines.

In order to determine whether DAC treatment induces changes in the expression levels of ADAM17, we quantified ADAM17 expression on the surface of KG1a and NB4 cells before and after DAC treatment by flow cytometry. No changes in the expression of ADAM17 were detected after treatment with DAC for 48 hours (Figure 3B). Nonetheless, we found that DAC significantly reduced ADAM17 activity in untreated and DAC-treated AML cells (*p* = 0.01 for KG1a and *p* = 0.039 for NB4) when analyzed by a fluorometric assay (Figure 3C). Therefore, these findings suggest that the proteolytic activity of ADAM17, and not its expression, could be directly or indirectly damaged upon exposure of AML cells to demethylating treatments, leading to less shedding of NKG2DL.

### DAC modulates sMICA, sMICB and sULBP2 release through demethylation of TIMP3

**Figure 4:**
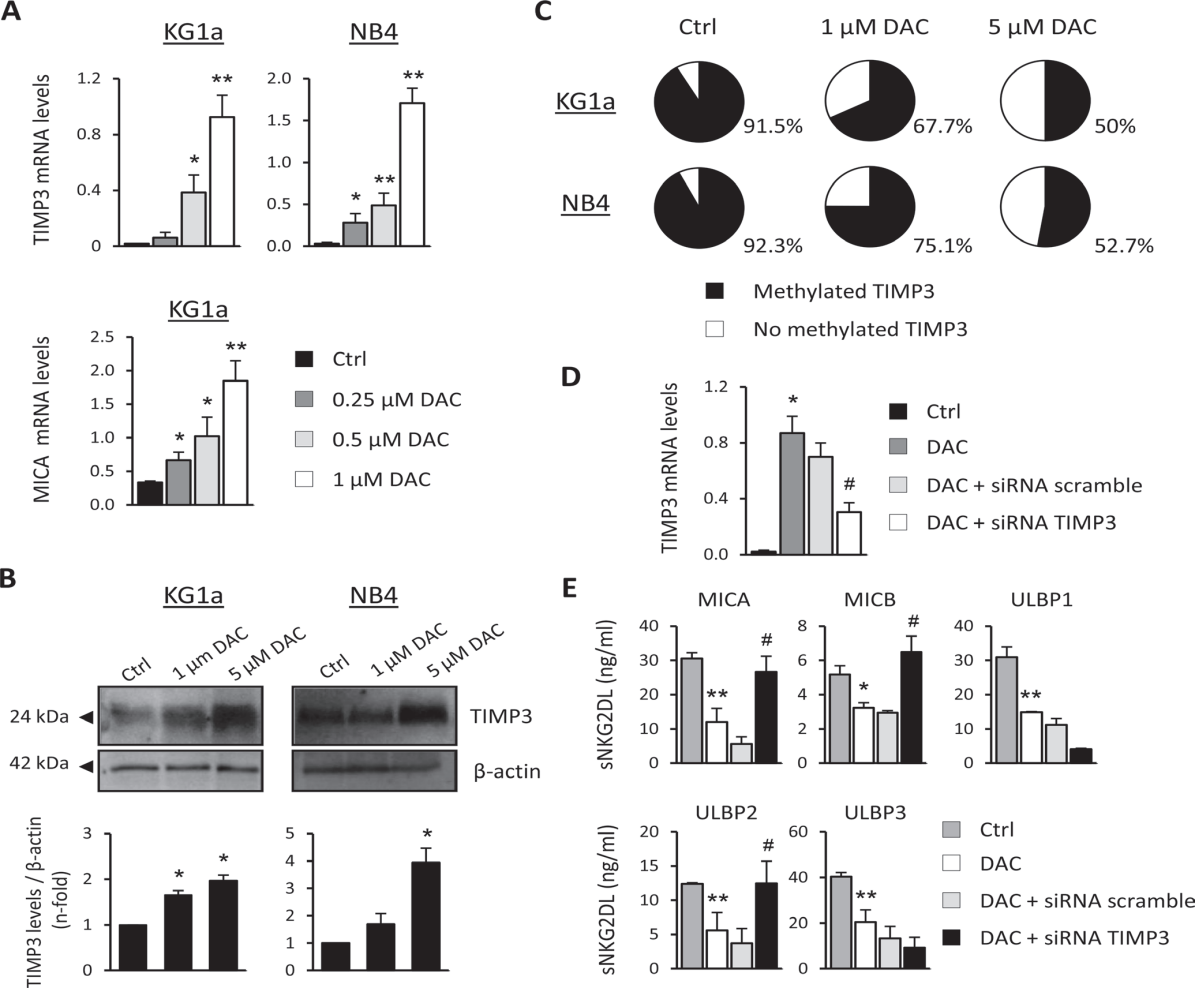
TIMP3 is involved in the release of sMICA, sMICB and sULBP2 in AML cell lines (**A**) KG1a and NB4 cells were treated with DMSO (Ctrl) or DAC (0.25, 0.5 and 1 μM) for 48 hours, and TIMP3 expression was analyzed by qRT-PCR. Each bar represents the relative expression of TIMP3 normalized with respect to the reference gene (GAPDH), using the 2^−ΔCt^ method. MICA transcription levels in the KG1a cell line untreated (DMSO, Ctrl) or treated with DAC at different concentrations were used as a positive control. Results are summarized as the mean ± SEM of five independent experiments. *p < 0.05 and ^*^p < 0.01. (**B**) TIMP3 protein levels were evaluated by western blot in KG1a and NB4 cells after treatment with DMSO (Ctrl) or DAC (1 μM or 5 μM) for 48 hours. *p < 0.05. (**C**) The TIMP3 methylation pattern was quantified by pyrosequencing in AML cell lines (KG1a and NB4 cells) before and after treatment with 1 μM or 5 μM DAC. Pie charts show the average percentage of methylation for the CpGs analyzed in the *TIMP3* gene. (**D**) TIMP3 expression was inhibited by transfection of KG1a cells previously treated with DAC (1 μM) with a TIMP3-specific siRNA or nonspecific scramble siRNA (200 nM). *p < 0.05 (**E**) Soluble NKG2DL were quantified by sandwich ELISA after TIMP3 inhibition. Values shown are the mean ± SEM of three independent experiments. * versus control and *p* < 0.05; ^*^ versus control and *p* < 0.01 ^#^ versus nonspecific scramble siRNA and *p* < 0.05.

The tissue inhibitor of metalloproteinase-3 (TIMP3) works as a natural inhibitor of ADAM17 and has also been shown to block the release of sNKG2DL [26]. First, we examined whether DAC treatment could induce changes in the expression of TIMP3. To this end, KG1a and NB4 cells were cultured for 48 hours with growing concentrations of DAC and TIMP3 expression was analyzed by qRT-PCR and western blot. While the TIMP3 transcription levels were very low or nearly absent at basal level in both AML cell lines, a significant dose-dependent increase in TIMP3 was observed after DAC treatment (Figure 4A). As a positive control, we analyzed the transcription levels of MICA in KG1a cells, which our group had previously reported to be highly methylated in this cell line. Moreover, these results are correlated with a clear increase in TIMP3 protein levels relative to basal conditions after DAC treatment in both cell lines (Figure 4B).

These findings led us to hypothesize that epigenetic mechanisms such as DNA methylation might regulate the expression of the *TIMP3* gene in AML cells. To find evidence to support this hypothesis, we determined the *TIMP3* DNA methylation profile by pyrosequencing before and after treating KG1a and NB4 cells with DAC. The analyzed region contains seven CpG sites within a CpG island located between positions +579 bp and +637 bp. Results show that the *TIMP3* gene is highly methylated in KG1a and NB4 cells (91.5% and 92.3%, respectively), whereas these levels were partially reduced (50% in KG1a and 52.7% in NB4) after treatment with DAC (5 μM, Figure 4C). The decrease in the DNA methylation level for the *TIMP3* gene was DAC dose-dependent, corroborating the specificity of the demethylating agent treatment. However, *TIMP3* was fully unmethylated in the HELR AML cell line, which did not release sNKG2DL (data not shown). These data suggest that demethylation of *TIMP3* could be associated with their increased expression levels in AML cells after DAC treatment.

Next, the direct involvement of TIMP3 in regulating and shedding sNKG2DL was confirmed by gene silencing (Figure 4D). KG1a cells were treated with DAC in order to increase TIMP3 expression, and further TIMP3 was silenced using small interfering RNA (siRNA). While treatment with DAC significantly reduces the levels of all NKG2DL in the cell-free supernatants, specific silencing of TIMP3 restores the shedding and release of soluble MICA (*p* = 0.001), MICB (*p* = 0.031) and ULBP2 (*p* = 0.002) (Figure 4E). However, the soluble ULBP1 and ULBP3 remain unchanged. Thus, the TIMP3 DNA methylation pattern could modulate the sheddase activity of ADAM17 and thereby the release of sMICA, sMICB and sULBP2. Additional mechanisms involved in the ADAM17 regulation and induced by hypomethylating agents could be responsible for the downregulation of the soluble levels for ULBP1 and ULBP3, although this aspect requires further analysis.

### Vidaza^®^-treated AML patients show reduced sNKG2DL release

Two hypomethylating agents (Vidaza^®^ and Dacogen^®^) are currently approved for the treatment of AML patients. Decitabine is indicated for those aged 65 years or more and who are not considered candidates for induction chemotherapy, while Vidaza^®^ is administered to AML patients as an initial regimen and to those who have relapsed. For these reasons, Vidaza^®^ is used more widely than decitabine in AML patients with high percentage of blasts (> 20%) or myelodysplastic related changes.

**Figure 5:**
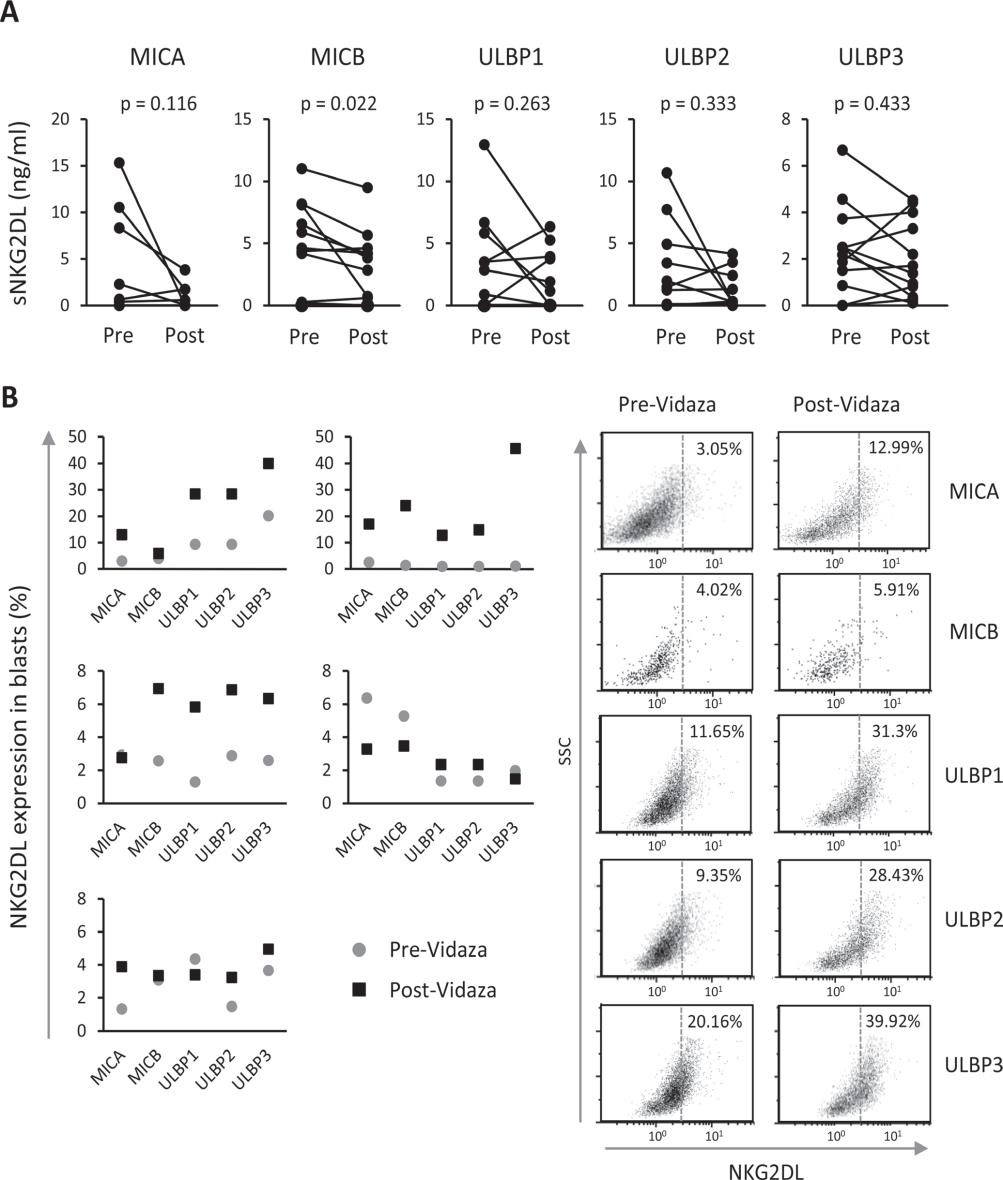
Vidaza^®^-treated AML patients show lower sNKG2DL levels and enhanced NKG2DL expression in blasts (**A**) Soluble NKG2DL were quantified by sandwich ELISA in sera from twelve AML patients before and after Vidaza^®^ treatment. Lines represent the levels of each sNKG2DL (ng/mL) before and after treatment of each individual AML patient. (**B**) Expression of NKG2DL on the cell surface of blasts from five AML patients before and after Vidaza^®^ treatment (left panel). The right panel shows dot plots of NKG2DL expression on the cell surface of blats from a representative patient. Numbers included in the figure quadrants indicate the percentage of positive cells for each NKG2DL.

We quantified the soluble levels of MICA, MICB and ULBPs1-3 by ELISA in the sera of twelve patients obtained before and after treatment with Vidaza^®^. All patients showed soluble levels of two or more NKG2DL. MICB and ULBP3 were the most commonly released NKG2DL (83.3% of the patients), followed by ULBP2 (66.7%), ULBP1 (58.3%) and MICA (50%) (data not shown). When we compared the soluble levels of each NKG2DL before and after treatment, we noted a clear tendency towards the reduction of sNKG2DL after treatment although not significant except for MICB (*p* = 0.022) (Figure 5A). More than 60% of the patients who were positive for some soluble ligand before treatment had lower levels of these ligands after Vidaza^®^ treatment.

We were only able to obtain blasts from the bone marrow of five patients before and after Vidaza^®^ treatment. There was a clear increase in the expression of some NKG2DL on the cell surface of blasts of three patients, whilst this increase was more discrete or nonexistent in the other two patients (Figure 5B). Patients who received the highest number of cycles (between 7 and 13) of Vidaza^®^ showed the greatest increase in NKG2DL expression (data not shown).

**Figure 6:**
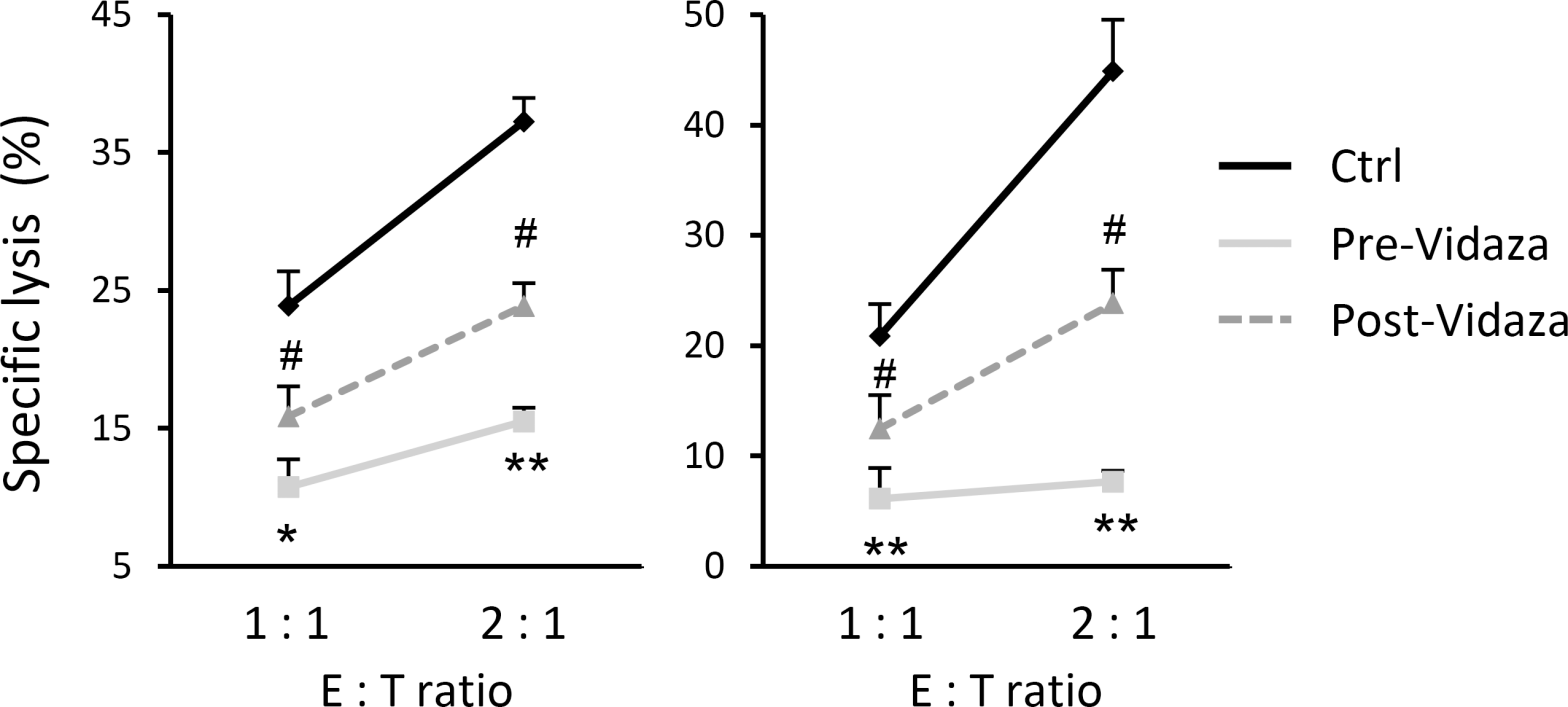
Treatment with Vidaza^®^ enhances the immune recognition mediated by NKG2D-NKG2DL engagement NKL cells were co-incubated with C1R-MICA transfectants at the indicated E:T ratios (1:1 and 2:1) in a cell lysis assay, in the presence of sera obtained before and after treatment with Vidaza^®^ from two independent AML patients. Values are the mean ± SEM of the percentage of specific lysis from three independent experiments. * versus control and *p* < 0.05; ^*^ versus control and *p* < 0.01 ^#^ versus pre-Vidaza^®^ sera and *p* < 0.05.

To determine the *in vivo* effect of the treatment with hypomethylating agents on immune recognition mediated by NK cells, we carried out a cytotoxicity assay using the NKL cell line as effector and C1R-MICA transfectants as target cells, at different E:T ratios. The co-culture assay was performed in the presence of serum from two independent AML patients obtained before and after Vidaza^®^ treatment. The specific lysis of target cells was significantly inhibited in the presence of pre-Vidaza^®^ sera (positive sNKG2DL) at all analyzed ratios (*p* = 0.006 and *p* = 0.01 at 2.1 E:T ratio for both patients respectively), and was partially restored after co-culture in the presence of post-Vidaza^®^ sera (low levels of sNKG2DL; *p* = 0.025 and = 0.017 01 at 2.1 E:T ratio for both patients) (Figure 6). These results suggest that treatment of AML patients with hypomethylating agents limits the release of soluble NKG2DL in sera, contributing to a stronger immune recognition by NKG2D-positive cytotoxic cells.

### Aberrant *TIMP3* DNA methylation is associated with an adverse cytogenetic prognosis

**Figure 7:**
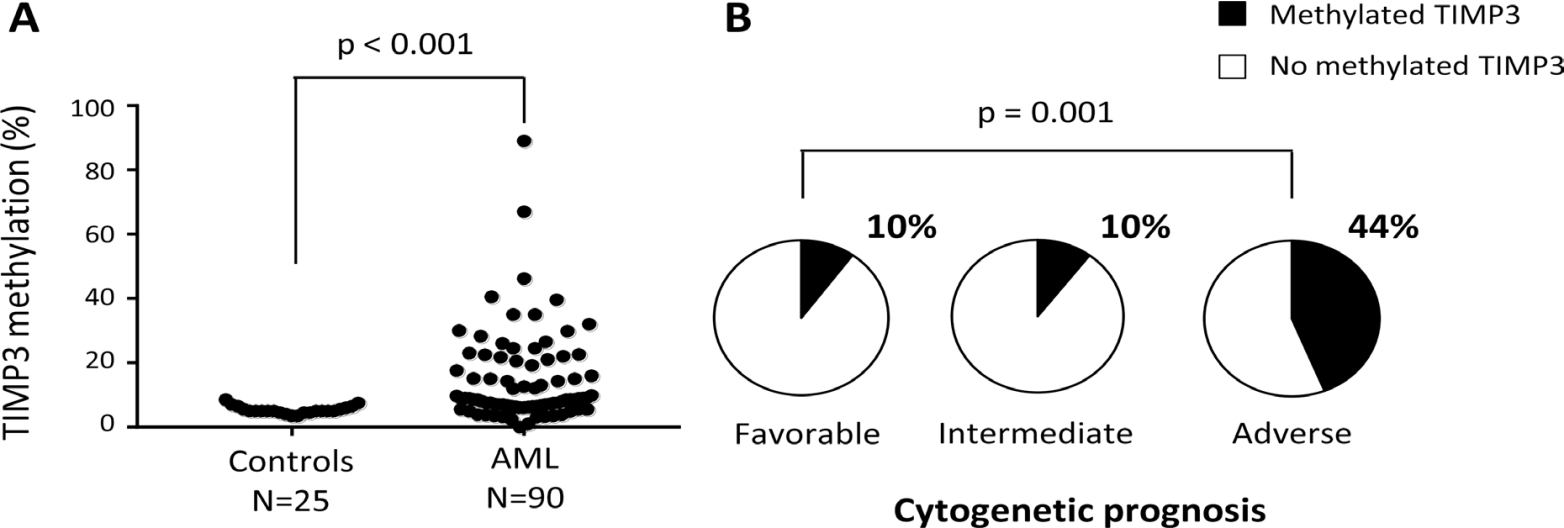
Aberrant TIMP3 DNA methylation levels in AML patients are associated with an adverse cytogenetic prognosis (**A**) The *TIMP3* DNA methylation levels were quantified by pyrosequencing in bone marrow cells obtained at diagnosis from 90 AML patients and from 25 healthy donors. Each sample is represented by a black dot. (**B**) Pie charts indicating the frequency of AML patients with aberrant DNA methylation levels (> 20%) for *TIMP3* by cytogenetic prognosis group (favorable, intermediate and adverse) of AML patients.

To confirm the results previously obtained in AML cell lines, the *TIMP3* DNA methylation pattern was analyzed in bone marrow cells obtained from 90 AML patients and 25 healthy donors, using the bisulfite pyrosequencing technique. The *TIMP3* DNA methylation levels observed in healthy donors were highly homogeneous (median: 5 ± 1.2%, range 0 – 8.5%) whilst AML patients show highly variable methylation rates for *TIMP3* (median: 8.8 ± 13.9%; range: 0–89%) (Figure 7A). Comparative analysis showed TIMP3 to be significantly methylated in AML patients compared with healthy donors (*p* < 0.001). Taking a DNA methylation rate greater than 20% to be anomalous, we found that 25.5% (23/90) of the AML patients exhibited aberrant DNA methylation for *TIMP3*.

To investigate the association between *TIMP3* DNA methylation and the clinical characteristics of AML patients, we classified the patients as unmethylated (TIMP3 DNA methylation levels < 20%) and methylated (TIMP3 methylation rate ≥ 20%). No statistically significant association was found with age, sex, FAB classification, AML etiology (primary or secondary) or induction therapy (data not shown). In order to analyze the genetic prognosis, AML patients were divided into three groups (favorable, intermediate and adverse cytogenetic prognosis) based on the abnormalities described by Yohe et al. [29]. Data show a significant association between DNA methylation status for *TIMP3* and cytogenetic prognosis (*p* = 0.001) (Figure 7B). AML patients harboring methylated *TIMP3* had a higher frequency of the adverse karyotype (44%) than did those with a favorable or intermediate prognosis (10% each). We also observed that 80% of AML patients with trisomy 11 and 66.7% with deletions linked to TP53 gene loss showed aberrant methylation for *TIMP3* (Table 1). Interestingly, mutations in the FMS-like tyrosine kinase (FLT3) gene, specifically internal tandem duplications (FLT3-ITD), were clearly associated with high levels of TIMP3 methylation (*p* = 0.002) (Table 2). Therefore, aberrant *TIMP3* DNA methylation in AML patients could be associated with an adverse genetic and cytogenetic prognosis, specifically with the presence of trisomy 11 and deletions linked to TP53 pro-oncogene loss.

**Table 1:** Genetic alterations of AML patients in association with TIMP3 methylation

Variant	Methylated TIMP3 *n* = 23 (%)	Unmethylated TIMP3 *n* = 67 (%)	*p*
**Cytogenetic characteristics**			
t (8;21)	1 (20%)	4 (80%)	ns
t (15;17)	1 (7.7%)	12 (92.3%)	ns
inv (16) or t (16;16)	1 (10%)	9 (90%)	ns
t (9;11)	0 (0%)	2 (100%)	ns
−7/−7q	1 (25%)	2 (75%)	ns
−21	0 (0%)	5 (100%)	ns
+4	0 (0%)	3 (100%)	ns
+8	2 (33.3%)	4 (66.7%)	ns
+11	4 (80%)	1 (20%)	0.001
+13	0 (0%)	2 (100%)	ns
+21	1 (20%)	4 (80%)	ns
del5q	3 (37.5%)	5 (62.5%)	ns
del7q	2 (28.6%)	5 (71.4%)	ns
del17p (p53)	4 (66.7%)	2 (33.3%)	0.007
Normal karyotype	2 (8.7%)	21 (91.3%)	ns
Complex karyotype	3 (42.8%)	4 (57.2%)	ns
Gene mutations			
NPM1	5 (41.7%)	7 (58.3%)	ns
FLT3-D835	1 (12.5%)	7 (87.5%)	ns
FLT3-ITD	6 (75%)	8 (25%)	0.001
CEBPA	2 (66.7%)	1 (33.3%)	ns

ns: not significant.

**Table 2:** Clinical characteristics of AML patients

Characteristics	AML patients *n* = 90 (%)
**Age (years)**	
< 60	44 (45.8%)
> 60	46 (54.2%)
**Sex**	
Male	52 (57.8%)
Female	38 (42.2%)
**FAB classification**	
M0	4 (4.5%)
M1	6 (6.7%)
M2	28 (31.1%)
M3	12 (13.3%)
M4	9 (10%)
M5	23 (25.5%)
M6	0 (0%)
M7	5 (5.6%)
Unclassified	3 (3.3%)
**Type of AML**	
Primary	60 (66.4%)
Secondary to SMD	15 (16.7%)
Secondary to other cancer	15 (16.7%)
**Cytogenetic prognosis**	
Favorable	29 (32.2%)
Intermediate	20 (22.2%
Adverse	41 (45.6%)
**Therapy**	
Chemotherapeutic	42 (46.7%)
Palliative care	14 (15.5%)
Allo-TPH	18 (20%)
Auto-TPH	10 (11.1%)
Missing-data	6 (6.7%)

## DISCUSSION

The highly heterogeneous nature of AML makes it a difficult disease to treat effectively. The hypomethylating agents azacitidine and decitabine are currently approved for AML patients who are ineligible for allogeneic stem cell transplantation and induction chemotherapy [30]. Considerable efforts have been made to identify the targets where these drugs exert their function. NKG2D, the main activating receptor of NK cells, plays an important role in the immune recognition of AML blasts through interaction with their NKG2DL (MICA, MICB and ULBPs1-3). However, AML cells have developed several mechanisms to evade this recognition, such as NKG2DL release from the cell surface in a soluble form that promotes NKG2D internalization and immune evasion [31]. Therefore, it is of great interest to understand the effect of the hypomethylating agents in the release of sNKG2DL by AML cells and on NKG2D-NKG2DL-mediated recognition. Here, we show that treatment with the hypomethylating agents limits the release of sNKG2DL by AML cells through the inhibition of ADAM17 activity. For MICA, MICB and ULBP2, we demonstrated that blockage of the shedding mediated by ADAM17 is caused by the demethylation of their inhibitor TIMP3. Thus, the use of these hypomethylating drugs could be useful for enhancing the immune recognition mediated by NKG2D-NKG2DL in AML patients.

First, we observed that hypomethylating treatment significantly reduced the release of the soluble form of all NKG2DL (MICA, MICB, ULBPs1-3), and enhanced their expression on the surface of KG1a and NB4 cells. Cell-free supernatants obtained from untreated AML cell lines, which show high levels of soluble NKG2DL, downregulate the expression of the NKG2D receptor, diminishing recognition and lysis by NKL cells. By contrast, supernatants from AML cells treated with DAC have lower soluble levels of these ligands, increased expression on the cell surface and thereby a higher level of recognition by cytotoxic cells.

Analysis of soluble NKG2DL in serum from AML patients before and after Vidaza^®^ treatment confirmed that soluble levels of all NKG2DL are diminished after treatment. Preliminary data from this study suggest that NKG2DL expression on the cell surface of AML blasts could be enhanced in proportion to the number of cycles of hypomethylating agent received, but we are aware of the limitation of this analysis due to the small number of cases involved. This is the first demonstration of hypomethylating agents controlling the release of sNKG2DL in AML cells. However, further analysis with a larger cohort of patients and samples is needed to verify the functional *in vivo* consequences of these treatments on the release of NKG2DL from AML blasts.

The ADAMs family sheddases, ADAM10 and ADAM17, have been implicated in the proteolytic cleavage of NKG2DL in several types of cancer cells [20, 32–33]. Treatment of AML cell lines (KG1a and NB4) with an ADAM10-specific inhibitor (GI254023X) did not affect the release of sNKG2DL. However, ADAM17-specific inhibitors (GW280264X and TMI-1) significantly restricted the release of all NKG2DL, including sULBP1 and sULBP3. MICA, MICB and ULBP2 are mainly released by MMPs, while ULBP1 and ULBP3 are recruited and released in exosomes [17]. However, these mechanisms are not exclusive and, for example, ULBP3 can be released by the protease ADAM10 in Hodgkin lymphoma cells [34]. Moreover, some metalloproteases such as ADAM17 can be found in exosomes, where they can be active and release their substrates [24]. Recently, release of ULBP1 from the cell surface has been found to be associated with their endocytosis and proteosomal degradation [35]. Thus, the two mechanisms involved in the shedding of NKG2DL could interact or be dependent on the cellular context, the expression of the different metalloproteases or the trafficking properties of each NKG2DL.

It has been reported that ADAM17 is expressed by cell lines and primary AML cells [36]. Consistent with this, we found that KG1a and NB4 cells express ADAM17 on the cell surface and that this expression is not affected by DAC treatment. Nevertheless, ADAM17 activity was significantly reduced after treatment with the hypomethylating agent, which implies that decitabine may regulate the shedding of sNKG2DL through the modulation of ADAM17 activity. Understanding the mechanisms involved in ADAM17 functionality could be relevant for developing new treatments for AML patients.

Several studies have reported diminished expression of TIMP3, a natural inhibitor of ADAM17 activity, contributing to the invasion and migration of tumor cells of various types of epithelial cancers (e.g., thyroid and lung cancers, melanoma, etc.) [37–39]. Consistent with this, we observed that TIMP3 expression in AML cell lines was very low, but increased after treatment with DAC, suggesting that this gene could be suppressed by epigenetic mechanisms such as DNA methylation. Changes in the DNA methylation status of various oncogenes or tumor suppressor genes occur frequently during the development of AML. So, mutations in genes encoding enzymes involved in the regulation of DNA methylation such as DNMT3A, IDH or the TET family are frequently found in AML patients [40]. The *TIMP3* gene is hypermethylated in KG1a and NB4 cells, which show high levels of sNKG2DL, but is completely unmethylated in HELR cells, which do not release soluble ligands. Thus, the TIMP3 methylation rate could help modulate the release of soluble ligands in AML. Further, AML patients (25.5%) showed aberrant DNA methylation for *TIMP3* in blasts obtained from their bone marrows. *TIMP3* DNA methylation has been described in several epithelial cancers [41, 42], but this is the first time it has been found to be methylated in AML patients. Additionally, in this study, we show that specific silencing of TIMP3 after treatment with DAC partially restores the soluble levels of MICA, MICB and ULBP2, whereas sULBP1 and sULBP3 levels remain unmodified. Thus, demethylation of TIMP3 could be the main mechanisms involved in downregulating the shedding of MICA, MICB and ULBP2 in DAC-treated AML blasts. However, additional mechanisms should be sought to explain the reduced levels of sULBP1 and sULBP3 attained after treatment with hypomethylating agents and mediated by the sheddase ADAM17. Independently of TIMP3, ADAM17 can be regulated by other mechanisms, such as those involving miRNAs. Thus, it has been reported that miRNA-145, which is aberrantly methylated in various cancers, negatively regulates the expression of ADAM17 in renal and hepatocellular carcinomas [43, 44]. Moreover, other proteins such as the aminopeptidase N/CD13 and the membrane-anchored glycoprotein RECK, which are regulated by DNA methylation, negatively regulate ADAM17 activity by internalization or direct physical interaction, respectively [36, 45].

Most AML patients show some chromosomal anomality or gene mutations [46]. Although mutations of *FLT3*, *NPM1* and *CEBPA* are customarily found in AML, others have emerged in recent years that allow patients to be categorized on the basis of their evolution [29]. Here, we found that aberrant methylation levels in specific genes, in addition to abnormal mutations, could help stratify AML patients and establish a more accurate therapy. In AML patients, aberrant *TIMP3* methylation was significantly associated with an adverse cytogenetic prognosis, whilst methylated *TIMP3* was less prevalent in patients with a favorable or intermediate cytogenetic risk. FMS-like tyrosine kinase 3 (FLT3) is a member of the class III receptor tyrosine kinase (RTK) expressed in hematopoietic stem cells, which are involved in processes such as proliferation and cell activation [47]. We found that patients with high levels of TIMP3 methylation more frequently feature FLT3 mutations, two of which are clearly defined in AML patients: internal tandem duplications (ITDs) and a point mutation in the Asp 835 of the A-loop (D835) [48, 49]. The frequency of patients with aberrantly methylated *TIMP3* is higher in FLT3-ITD (75%) than in FLT3-D835 (12.5%) mutations. These results are consistent with the poor prognosis of patients with FLT3-ITD mutations [50]. Moreover, specific cytogenetic analysis revealed that high levels of TIMP3 methylation were also significantly associated with trisomy 11 and deletions linked to TP53 gene loss. These results are in accordance with earlier findings in which trisomy 11 and 17p (p53) deletions were associated with poor prognosis in AML [51–53]. Thus, the DNA methylation levels of *TIMP3* could be a new biomarker that helps establish the cytogenetic prognosis of the disease.

Nowadays, therapies based on alloreactive NK cell transplantation have been proposed to strengthen the immunological response of AML patients [54]. In other types of leukemias, as chronic myeloid leukemia (CML), NK cells are able to kill CD34+ CML cells in the presence of intact HLA inhibitory molecules [55]. However, it is well known that the ability of NK cells to kill autologous leukemic blasts is disrupted by the weak expression of natural cytotoxicity receptors such as NKG2D [56–58]. Overall, our results suggest that hypomethylating based treatments could maximize the immune recognition mediated by NK cells. Moreover, it has recently been reported that long-term culture of NK cells leads to TIMP3 over-expression [59]. Although we found no expression of TIMP3 by NKL cells (data not shown), it would be interesting to examine whether expression of TIMP3 induced by activated NK cells is an additional strategy by which downregulation of sNKG2DL may be induced by AML blasts.

In summary, our study demonstrates that treatment with hypomethylating agents diminishes the release of sNKG2DL, which in turn promotes NKG2D-NKG2DL engagement. This effect is mediated by TIMP3 demethylation, which inhibits the activity of ADAM17 sheddase, leading to a more limited release of sMICA, sMICB and sULBP2 from the cell surface of leukemic blasts. Although hypomethylating drugs promote the induction of several genes during oncogenesis, the association found between aberrant DNA methylation of TIMP3 and poor prognosis for patients with the disease suggests that the *TIMP3* methylation rate could be an additional useful new prognostic biomarker of disease in AML and open the door to new therapeutic approaches by potentiating NK cell anti-leukemic potential.

## MATERIALS AND METHODS

### Cell lines, reagents and treatments

The human myeloid leukemia KG1a and NB4 cells lines were purchased from the American Type Culture Collection and the Leibniz-Institute DSMZ GmbH (Germany), respectively. Cells were maintained in RPMI-1640 medium containing 10% heat-inactivated fetal calf serum in a 5% CO_2_ incubator at 37°C. Cells were cultured at a density of 0.4 × 10^6^ cells/ml with complete media supplemented with DAC (0.25, 0.5, 1 or 5 μM from Sigma-Aldrich, St Louis, MO), ADAM10 inhibitor (GI254023X, 50 μM; Sigma-Aldrich) or ADAM17 inhibitors (GW280264X at 10 μM; TMI-1 at 10 and 25 μM from Aobious Inc., Gloucester, MA, and Tocris, Bristol, UK, respectively) for 48 hours. A dose-response curve was derived for all drugs to identify the doses that did not affect cell viability (data not shown). Cell viability was determined by staining with 7AAD (Immunostep Inc., Salamanca, Spain).

### Patient samples

To analyze TIMP3 DNA methylation, bone marrow samples were obtained from 25 healthy donors and 90 AML patients at diagnosis (aged 54 ± 24.57 years; 52 males and 38 females) in Hospital Clinico Universitario Virgen de la Arrixaca and Hospital General Universitario Santa Lucía de Cartagena (Murcia, Spain). Patients had been diagnosed with AML during the previous 10 years (2006–2016) according to the morphological and cytochemical criteria of the French-American-British (FAB) classification. The clinical characteristics of the patients are summarized in Table 2. Soluble NKG2DL was studied in a cohort of twelve AML patients treated with Vidaza^®^ after diagnosis (aged 65 ± 12.9 years; 5 males and 4 females) at the Hospital Universitario Central de Asturias (Asturias, Spain). NKG2DL expression on the cell surface of blasts before and after Vidaza^®^ treatment at different cycles was studied in a cohort of five AML patients from Hospital Clínico San Carlos (Madrid, Spain). All patients and healthy donors gave their written informed consent, in accordance with the Principles of the Declaration of Helsinki. The local hospitals’ ethics committees approved the study.

### Flow cytometry

AML cell lines (KG1a and NB4) were stained with the human-specific mAb for ADAM17 (Ref: MAB9301; clone #111633) or each NKG2DL: MICA (Ref: MAB1300; clone #159227), MICB (Ref: MAB1599; clone #236511), ULBP1 (Ref: MAB1380; clone #170818), ULBP2 (Ref: MAB1298; clone #165903) and ULBP3 (Ref: MAB1517; #clone 166510). All mAbs were from R&D Systems (Minneapolis, MN). Subsequently, cells were stained with FITC-goat anti-mouse immunoglobulin G (Biolegend, San Diego, CA) and analyzed with a Gallios™ flow cytometer (Beckman Coulter, Brea, CA, USA). In AML patients, blasts from bone marrow were stained with CD34-PE and CD45-APC antibodies (Biolegend). The specific fluorescence index was calculated by dividing the mean fluorescence obtained with the respective specific mAb by that of the secondary antibody.

### ELISA

Soluble NKG2DL (sNKG2DL) was detected using sandwich ELISA. Plates were coated with a specific mAb (4 μg/ml) for each NKG2DL. Coating mAbs are the same clones that we used for the previously described flow cytometry. Subsequently, plates were blocked with 2% BSA-PBS. After blocking, 100 μl of sera or supernatant samples and recombinant human (rh) Fc chimera proteins specific for each NKG2DL (rhMICA Ref: 1300-MA, rhMICB Ref: 1599-MB, rhULBP1 Ref: 1380-UL, rhULBP2 Ref: 1298-UL,and rhULBP3 Ref: 1517-UL all from R&D Systems) were added for 2 hours at RT. After washes, a specific biotinylated polyclonal antibody for each NKG2DL (0.4 μg/ml) (MICA Ref: BAF1300,, MICB Ref: BAF1599, ULBP1 Ref: BAF1380, ULBP2 Ref: BAF1298, and ULBP3 Ref: BAF1517, all from R&D Systems) was added for 2 hours at RT. Afterwards, plates were washed and incubated with streptavidin-HRP (GE Healthcare, Little Chalfont, UK). Plates were then washed and developed using TMB (tetramethylbenzidine; Sigma-Aldrich) and absorbance was measured at 450 nm. The detection threshold for each ELISA was less than 400 pg/ml and as a positive control we used the recombinant proteins specific for each ligand.

### Quantitative real-time PCR (qRT-PCR) analysis

Total cellular RNA was extracted with a GeneMATRIX Universal RNA purification kit (EUR_X_, Poland) according to the manufacturer's instructions. Reverse transcription of purified RNA was performed using the High-Capacity cDNA Reverse Transcription kit (Applied Biosystems, Foster City, CA). Next, quantitative gene expression analysis was performed by real-time PCR on a MyiQTM Single Color Real-Time PCR Detection System (Bio-Rad) using SYBR^®^ Green PCR Master Mix (Applied Biosystems). Data were normalized to GAPDH RNA expression and all samples were run in triplicate. Relative mRNA abundance was determined by comparison of threshold values and calculated by the 2-^ΔCT^ method (ΔCt: Ct gene test - Ct endogenous control). The following specific primers were used; TIMP3: fwd: 5′-TCCCAGCGCAAGGGGCTGAA-3′, rev: 5′-GCCGGATGCAGGCGTAGTGTT-3′; MICA: fwd: 5´-AATGGAACCTACCAGACCTGGG-3′, rev: 5´-AC ATGGAATGTCTGCCAATGACT-3´ and GAPDH: fwd: 5′-TCGGAGTCAACGGATTTGGTCGT3′; rev: 5′- TGCC ATGGGTGGAATCATATTGGA-3′.

### TACE activity assay

The activity of TACE (ADAM17) was determined with the Sensolyte 520 TACE Activity Assay Kit (AnaSpec, San Jose, CA) using 20 μg of cell lysate proteins, according to the manufacturer's protocol. This assay uses a 5-FAM (fluorophore) and QXL^®^ 520 (quencher) FRET peptide substrate. Upon cleavage of the FRET peptide by the active enzyme, the fluorescence of 5-FAM is recovered and monitored at an excitation of 490 nm and emission of 490/520 nm. Data are expressed as the mean fluorescence intensity per microgram of total protein.

### Small interfering RNA (siRNA) transfection

TIMP3 and control non-specific scrambled siRNA were purchased from Qiagen and Dharmacon (Denver, CO), respectively. 2 × 10^6^ cells were plated in 12-well plates and transfected with control or 200 nM of TIMP3-specific siRNA. Transfection was performed using the Cell Line Nucleofector^®^ Solution V and the AMAXA nucleofector apparatus (Lonza, Walkersville, MD). NKG2DL shedding was analyzed by ELISA 48 hours after transfection.

### Western blot

AML cell lines (KG1a and NB4) were lysed in RIPA buffer (Cell Signaling Technology Inc., MA) supplemented with protease inhibitors cocktail (Sigma-Aldrich). Samples were clarified by centrifugation, and stored at −20°C. 20 μg of protein extract were separated on 15% polyacrylamide-SDS gels and blotted onto nitrocellulose membranes. After blocking, primary polyclonal goat anti-human TIMP3 and ß-actin (Santa Cruz Biotechnology, Dallas, TX) were incubated overnight at 4°C. Membranes were washed and subsequently incubated with horseradish peroxidase-conjugated rabbit anti-goat IgG (Dakocytomation, Glostrup, Denmark) for 1 hour at room temperature. The blots were developed with the use of a chemiluminescent substrate (ECL Western Blotting Analysis System; Amersham Biosciences, Little Chalfont, UK).

### Bisulfite treatment and pyrosequencing

Firstly, genomic DNA was extracted using a DNeasy^®^ Blood & Tissue kit (Qiagen, Valencia, CA). 1 μg of purified DNA was modified with bisulfite using a DNA Methylation-Gold™ Kit (Zymo Research, Irvine, CA). Modified bisulfite DNA was amplified using specific biotinylated primers for TIMP3 designed by PyroMark Assay Design Software 2.0 (Qiagen). Primer sequences were as follows: fwd: 5′-TGGGTGGGTGTTAGTTGG-3′; rev: 5′-CCCCCTCAAACCAATAAC-3′; seq: 5′-ATTTA GTGGTTTAGGTGG-3′. Pyrosequencing was performed with the PyroMark™ Q24 System version 2.0.6, and methylation levels were quantified using PyroMark Q24 Software 2.0 following the manufacturer's instructions (Qiagen).

### Cytotoxicity assay

Cytotoxicity was assayed by flow cytometry using the PKH67 Green Fluorescent Cell Linker kit (Sigma-Aldrich). We used the NKL cell line as the effector [60] and the erythroleukemia K562 and C1R-MICA transfectants as target cells [61]. NB4 and KG1a cells were treated beforehand with DMSO or DAC (5 μM) for 48 hours and supernatants were collected. Target cells were previously cell membrane labeled with PKH67 dye (1 μM for 5 minutes) and further co-cultured with the NKL effector cells in the presence of cell-free supernatants from untreated or DAC-treated AML cells for 4 hours at 37°C. In blocking experiments, NKL cells were incubated beforehand with 10 μg/ml anti-human NKG2D blocking mAb (Biolegend) for 1 h at 37°C. For patient cytotoxicity assays, sera from two AML patients before and after Vidaza^®^ treatment were used. Cells were then stained with 5 μl of 7AAD and analyzed by flow cytometry. For gating strategy (Supplementary Figure 2), target cells were selected from effector cells by PHK67-positive staining, and further we analyzed the percentage of 7AAD-positive cells within the target cells (dead target cells). Dead cells from the negative control (target cells incubated in the absence of effector cells) are considered as spontaneous lysis. Percentage specific lysis was calculated as 100 × [(% dead target cells–% spontaneous lysis)/(100–% spontaneous lysis)].

### Statistical analysis

Data of soluble NKG2DL are expressed as mean ± standard error of the mean (SEM). All *in vitro* experiments were done at least three times with similar results. Differences between groups were examined using Student's paired-samples *t*-test and the Mann-Whitney *U* test. Comparison between the sNKG2DL levels from the AML patients sera before and after Vidaza treatment was analyzed using Wilconxon test. The chi-square test was used to compare the TIMP3 methylation status with the clinical characteristics in AML patients. In all cases, statistical significance was concluded for values of *p* < 0.05. Analyses were performed with SPSS version 15.0 (Chicago, IL, USA).

## SUPPLEMENTARY MATERIALS FIGURES


