# Correction: Increasing TIMP3 expression by hypomethylating agents diminishes soluble MICA, MICB and ULBP2 shedding in acute myeloid leukemia, facilitating NK cell-mediated immune recognition

**DOI:** 10.18632/oncotarget.26079

**Published:** 2018-08-28

**Authors:** Aroa Baragaño Raneros, Alfredo Minguela, Ramon M. Rodriguez, Enrique Colado, Teresa Bernal, Eduardo Anguita, Adela Vasco Mogorron, Alberto Chaparro Gil, Jose Ramon Vidal-Castiñeira, Leonardo Márquez-Kisinousky, Paula Díaz Bulnes, Amelia Martinez Marin, Maria Carmen García Garay, Beatriz Suarez-Alvarez, Carlos Lopez-Larrea

**Affiliations:** ^1^ Department of Immunology, Hospital Universitario Central de Asturias, Oviedo, Spain; ^2^ Immunology Service, Instituto Murciano de Investigación Biosanitaria (IMIB), Hospital Clínico Universitario Virgen de la Arrixaca, Murcia, Spain; ^3^ Department of Hematology, Hospital Universitario Central de Asturias, Oviedo, Spain; ^4^ Hematology Department, Hospital Clínico San Carlos, Instituto de Investigación Sanitaria San Carlos (IdISSC), Department of Medicine, Universidad Complutense de Madrid (UCM), Madrid, Spain; ^5^ Hematology Service, Hospital Clínico Universitario Virgen de la Arrixaca, Murcia, Spain; ^6^ Hematology Service, Hospital General Universitario Santa Lucía, Cartagena, Murcia, Spain

**This article has been corrected:** The correct author name is given below:

**Alfredo Minguela**

Original article: Oncotarget. 2017; 8:31959-31976. https://doi.org/10.18632/oncotarget.16657

