# Supplementary material for: Increasing TIMP3 expression by hypomethylating agents diminishes soluble MICA, MICB and ULBP2 shedding in acute myeloid leukemia, facilitating NK cell-mediated immune recognition
Source: Oncotarget. 2017 Mar 29;8(19):31959–76. doi: 10.18632/oncotarget.16657 (PMC5458262; doi:10.18632/oncotarget.16657)
Supplement: Supplementary file 1 [file oncotarget-08-31959-s001.pdf]

Increasing TIMP3 expression by hypomethylating agents diminishes soluble MICA, MICB and ULBP2 shedding in acute myeloid leukemia, facilitating NK cell-mediated immune recognition

Supplementary Materials

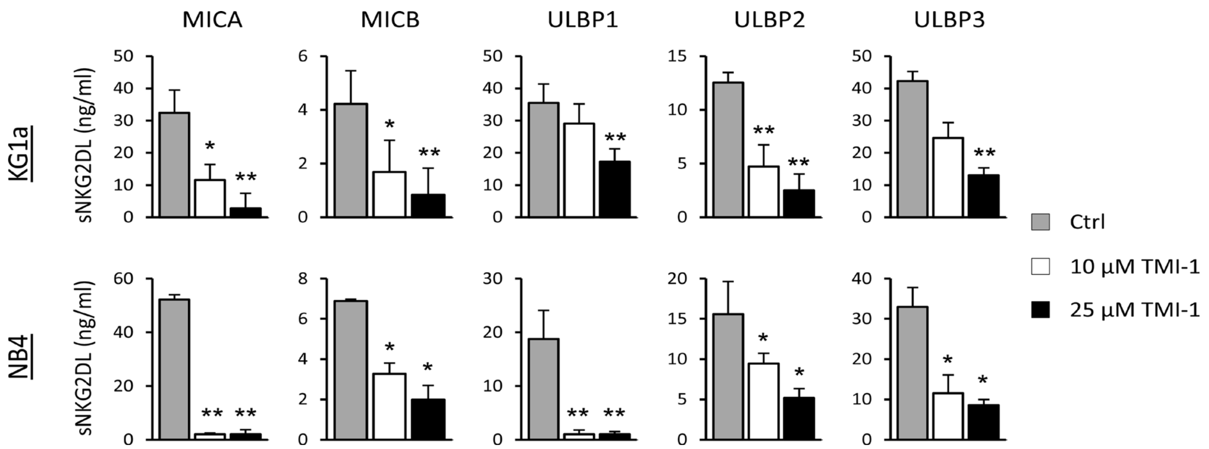

Supplementary Figure 1: ADAM17 modulates the release of sNKG2DL in AML cells. KG1a and NB4 cells were treated with an inhibitor specific to ADAM17 (10 or 25 µM of TMI-1) for 48 hours. Levels of soluble NKG2DL (sMICA/B and sULBPs1-3) were quantified by sandwich ELISA. Values are the mean ± SEM of at least three independent experiments. $^{*}p < 0.05$ and $^{**}p < 0.01$.

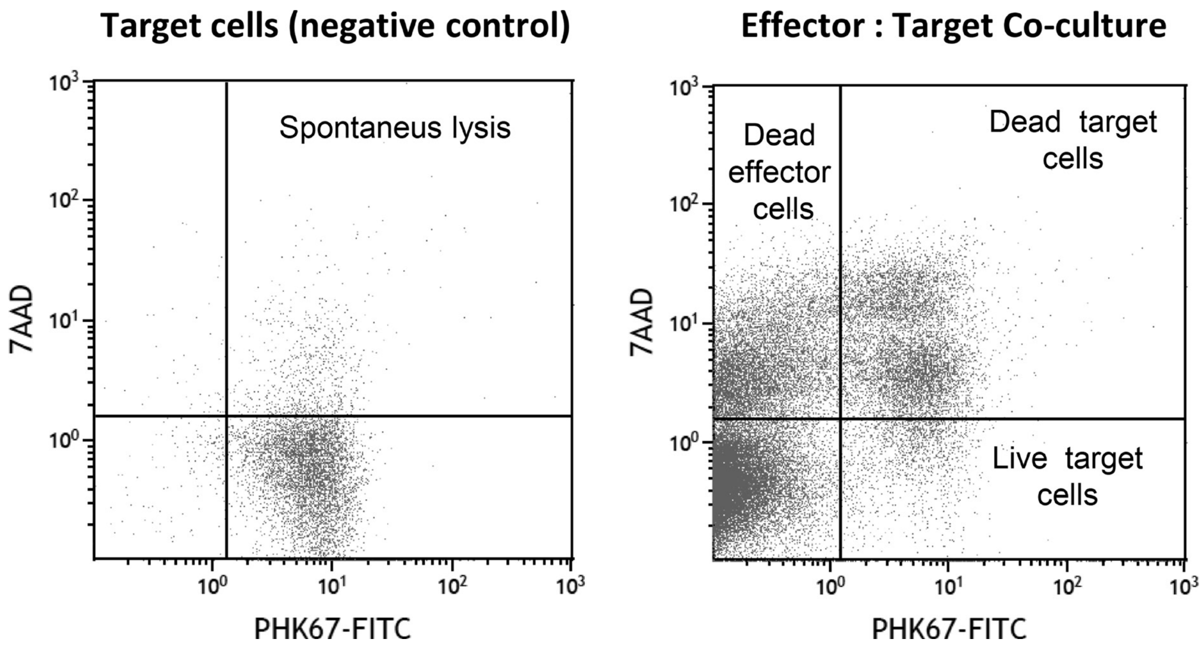

Supplementary Figure 2: Gating strategy followed in the cytotoxicity assays. Target cells were previously cell membrane labeled with PKH67 dye (1 μM for 5 minutes) and further co-cultured with the NKL effector cells for 4 hours under the conditions appropriate for each experiment. Cells were then collected and stained with the 7AAD viability solution. The cytotoxic capacity was analyzed by flow cytometry, selecting the PHK67-labeled target cells which are positive for 7AAD (dead target cells). Dead cells from the negative control (only target cells) are considered as spontaneous lysis. Percentage specific lysis was calculated as 100 × [(% dead target cells–% spontaneous lysis) / (100–% spontaneous lysis)].
